# Supplementary material for: A discovery platform for identification of host-induced bacterial biosensors from diverse sources
Source: Mol Syst Biol. 2025 Jun 9;21(9):1237–62. doi: 10.1038/s44320-025-00123-3 (PMC12405535; doi:10.1038/s44320-025-00123-3)
Supplement: Supplementary file 1 — Appendix [file 44320_2025_123_MOESM1_ESM.pdf]

Appendix Materials for

A discovery platform accelerates identification of host-induced bacterial biosensors from diverse sources.

Clare M. Robinson, David Carreño, Tim Weber, Yangyumeng Chen and David T. Riglar*

*Corresponding author. Email: d.riglar@imperial.ac.uk

Contents:

Figure	Page
Appendix Figure S1	2
Appendix Figure S2	3
Appendix Figure S3	4
Appendix Figure S4	5

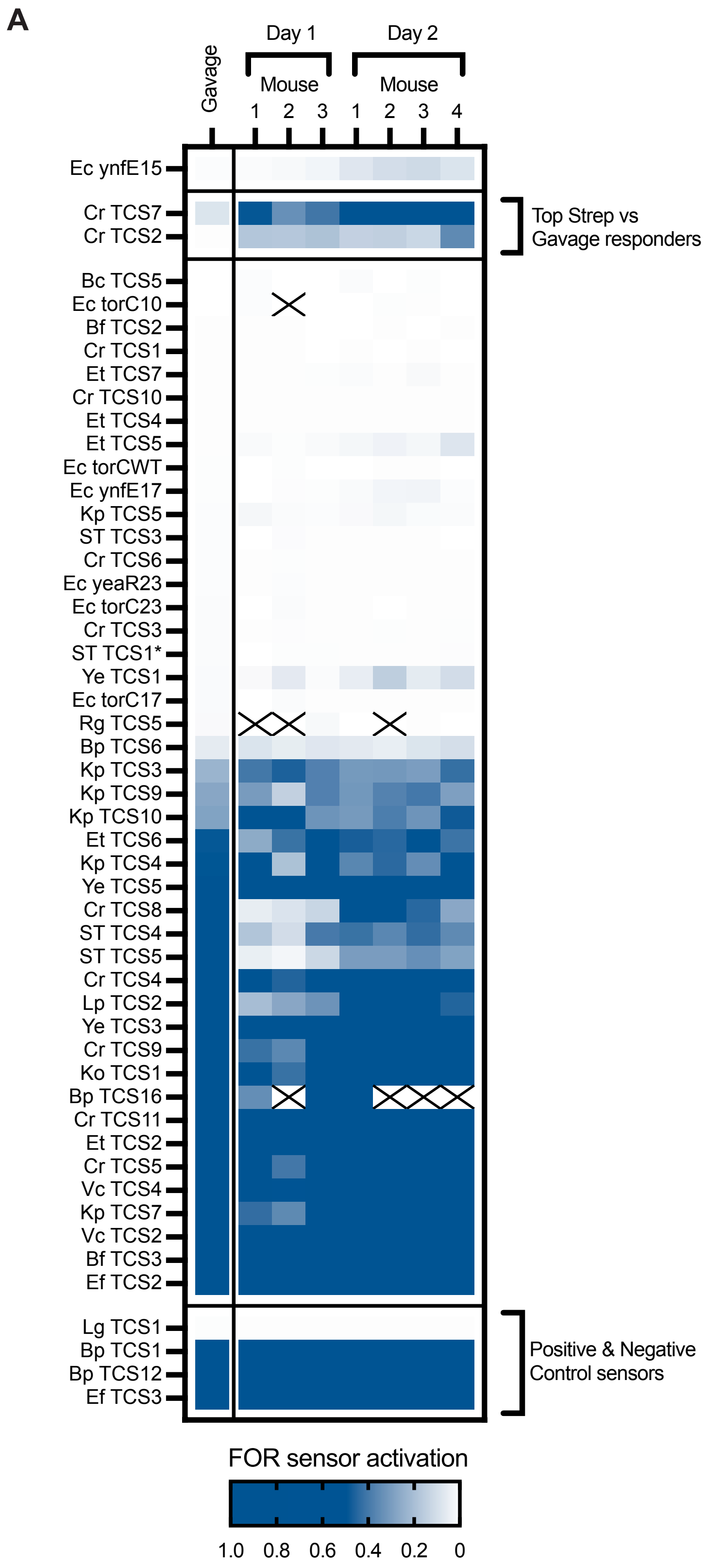

Appendix Figure S1: Library 1 sensor response to the murine gut environment. A) Sensor activation (FOR) of all sensors in all datapoints passing QC on days 1 (n=3) and day 2 (n=4). Heatmap shows the median FOR of all barcodes for each sensor. Squares marked X indicate insufficient sequencing read data for that sensor and condition.

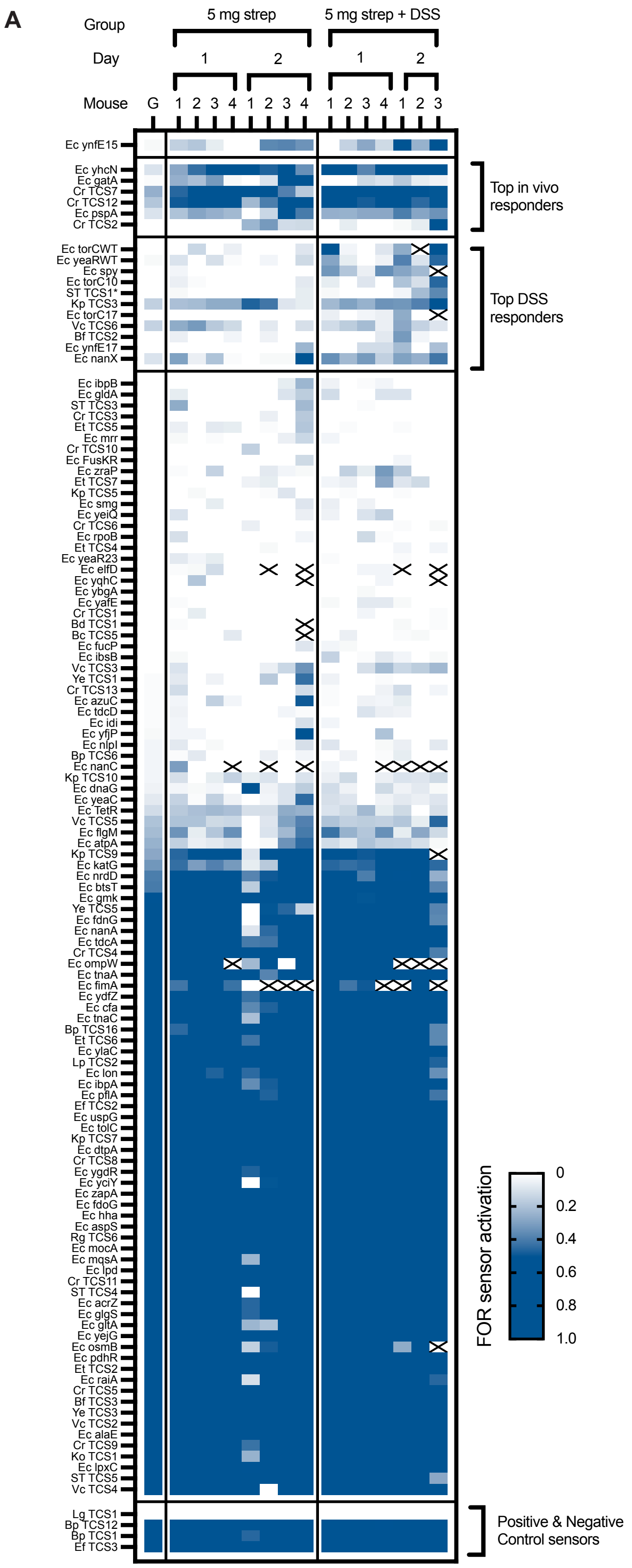

Appendix Figure S2: Combined Library 1 + 2 sensor response to the murine gut with and without induced inflammation A) Full biosensor activation data for all Library 1 and 2 sensors across all mice and timepoints passing QC criteria (n=4 for both 5mg Strep Control days and Day 1 5mg Strep + DSS; n=3 for Day 2 5mg Strep + DSS). Heatmap shows median FOR from pooled barcodes for each sample. Squares marked X indicate insufficient sequencing read data for that sensor and condition.

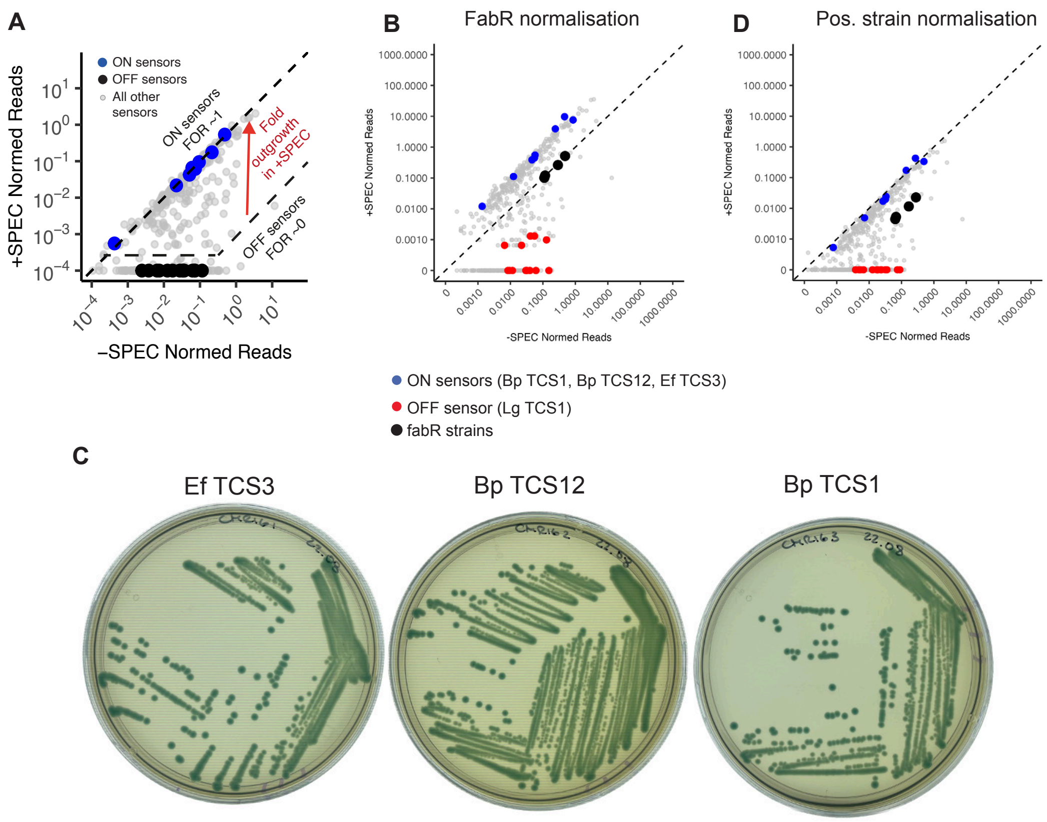

Appendix Figure S3: Identification of internal Positive Normalisation Strains for Odds ratio calculation A) Normalised reads of a typical sample, with ON and OFF control sensors highlighted. ON sensors will have equal growth in the presence (+SPEC) or absence (-SPEC) of spectinomycin selective media, resulting in a fractional odds ratio (FOR) of ~1. OFF sensors will not grow in spectinomycin selective media (+SPEC), resulting in a FOR of ~0. B) Example data normalised using spiked in Ec fabR controls. C) Individually cloned new positive control strains (Ef TCS3, Bp TCS12, and Bp TCS1) streaked on X-gal indicator plates demonstrated 100% memory-ON. D) Example data using normalisation with internal positive control strains.

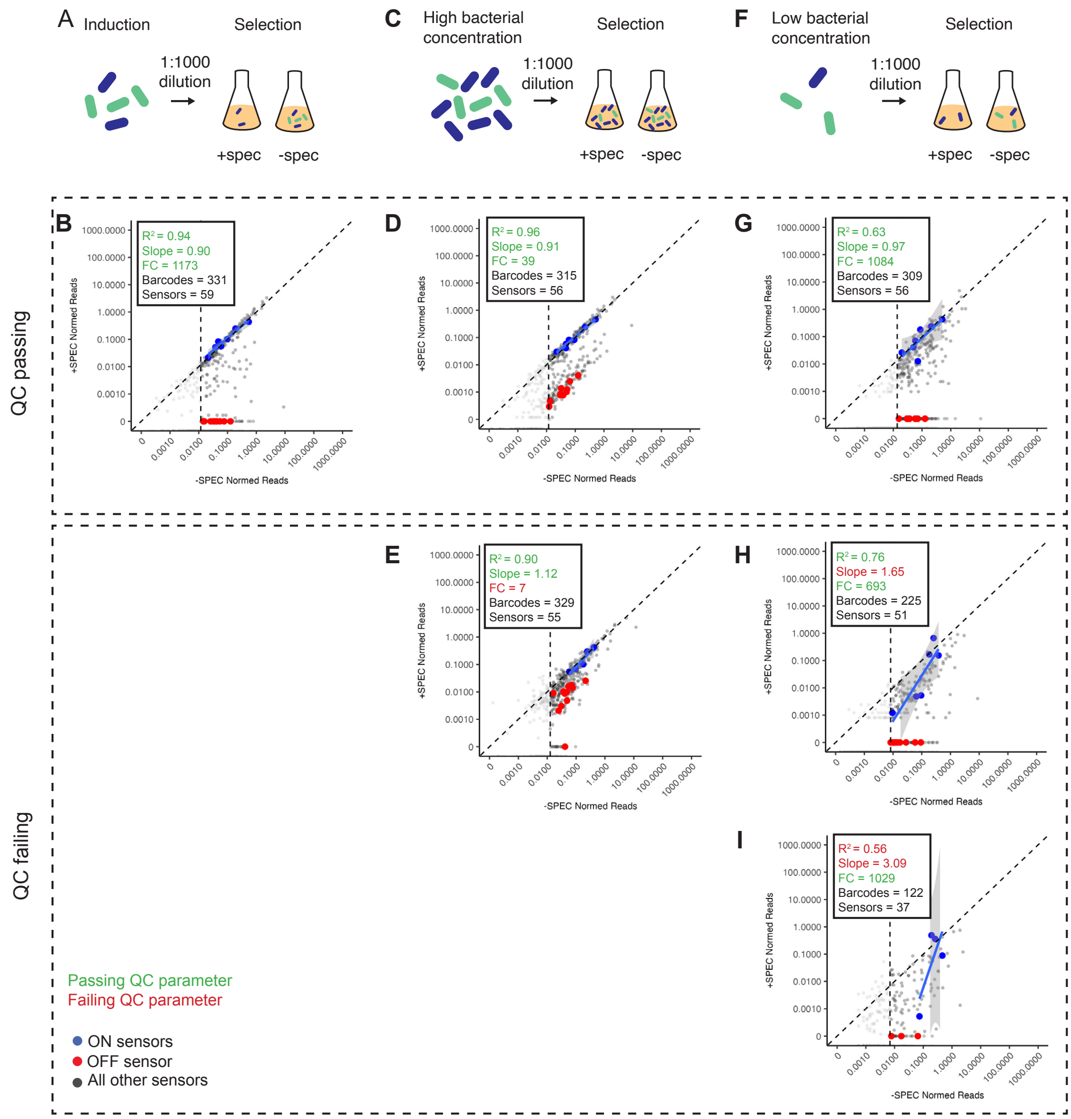

Appendix Figure S4: Quality Control parameters A-B) Optimal sample conditions, in which there is a high fold-change between positive and negative strains, and positive strains show low outgrowth variability. C-E) High bacterial concentration is assumed to lead to a lack of outgrowth potential of ON strains in +SPEC samples, leading to lower fold-change between positive and negative controls strains. F-I) Low bacterial concentration at the dilution and selection stage is assumed to lead to variability in outgrowth of positive and activated strains, leading to greater variation in sensor activation distributions. QC failing samples (E), (H) and (I) have QC failing parameters marked in red.
