# Supplementary material for: A discovery platform for identification of host-induced bacterial biosensors from diverse sources
Source: Mol Syst Biol. 2025 Jun 9;21(9):1237–62. doi: 10.1038/s44320-025-00123-3 (PMC12405535; doi:10.1038/s44320-025-00123-3)
Supplement: Supplementary file 2 — Table EV1 [file 44320_2025_123_MOESM2_ESM.docx]

**Table EV1:** Primers and DNA fragments used in this study

| **Name** | **Sequence** |
| --- | --- |
| Ultramer DNA Barcode cassette | ATGCTCTTCTAGATCGTCGGCAGCGTCAGATGTGTATAAGAGACAGNNNNNNNNNNNNNNNNNNNNNNNNNNNNNNNNNNNNNNNNNNNNNNNNNNNNNNNNNNNNNNNNNNNNNNNNNNNNNNNNNNNNNNNNNNNNNNNNNNNNNNNNNCTGTCTCTTATACACATCTCCGAGCCCACGAGACGAATGAAGAGCGG |
| p070_Barcode_cassette_F_duplexing_primer | CCGCTCTTCATTCGTCTCGT |
| p075_Barcode_cassette_R_duplexing_primer | ATGCTCTTCTAGATCGTCGGC |
| p079_Barcode_cassette_F_duplexing_primer_BsaI | CAGGTCTCAGAGGTCGTCGGCAGCGTCAGAT |
| p100_Barcode_cassette_R_duplexing_primer_BsaI | ACGGTCTCATCCGTGTCTCGTGGGCTCGGAGAT |
| p145_p5_site | TCGTCGGCAGCGTCAG |
| p146_p7_site | GTCTCGTGGGCTCGGAG |
| p117_Nissle_Lib_seq_new_F | CTCACTATAGAGGGACAACC |
| p118_Nissle_Lib_seq_new_R | AATGGTTTCTTTTTTGTGCTCAT |
| DTR304_Fwd | ATGCTCTTCTGAATGCAATACCTTAGCGGGCTTTA |
| DTR304_DT305_Rev | TAGCTCTTCACATCGCAAAAGTATCCTGAGGC |
| DTR305_Fwd | ATGCTCTTCTGAACATCTAACTCTTTGTGAAATAAATCAAA |
