# Supplementary material for: A discovery platform for identification of host-induced bacterial biosensors from diverse sources
Source: Mol Syst Biol. 2025 Jun 9;21(9):1237–62. doi: 10.1038/s44320-025-00123-3 (PMC12405535; doi:10.1038/s44320-025-00123-3)
Supplement: Supplementary file 3 — Table EV2 [file 44320_2025_123_MOESM3_ESM.docx]

**Table EV2:** Bacterial strains used in this study

|  | **E. coli parent strain** | **Sensor** | **Source/reference** |
| --- | --- | --- | --- |
| PAS811 | E. coli NGF-1 | None | (Naydich *et al.*, 2019) |
| DTR270-273 | PAS811 | *fabR* + barcode | This paper |
| DTR274-276 | PAS811 | *ynfE15* + barcode | This paper |
| DTR277-279 | PAS811 | *S.* Typhimurium ttrSR-pttrBCA (RBS) (ST TCS1*) | This paper |
| DTR280-283 | PAS811 | *hycAWT* + barcode | This paper |
| DTR284 | PAS811 | *torC23* + barcode | This paper |
| DTR285-288 | PAS811 | *torCWT* + barcode | This paper |
| DTR289-292 | PAS811 | *ynfE17* + barcode | This paper |
| DTR293 | PAS811 | *torC17* + barcode | This paper |
| DTR294-297 | PAS811 | *torC10* + barcode | This paper |
| DTR298 | PAS811 | *ST ttrSR-pttrBCA (wt)* + barcode | This paper |
| DTR299 | PAS811 | *Bp TCS1*+ barcode | This paper |
| DTR300 | PAS811 | *Bp TCS12* + barcode | This paper |
| DTR301 | PAS811 | *Ef TCS3* + barcode | This paper |
| DTR302 | PAS811 | *Et TCS7: E. tarda  ttrSR-pttrBCA* + barcode | This paper |
| DTR303 | PAS811 | *Cr TCS2: C. rodentium (ICC168) dpiAB (WP_012904972.1 and RR: WP_012904973.1) - P_citC_*  *(to gene locus tag ROD_RS03175)* + barcode | This paper |
| DTR304 | PAS811 | *Truncated Cr TCS2: ΔdpiAB* + barcode | This paper |
| DTR305 | PAS811 | *Truncated Cr TCS2: ΔdpiAB , ΔdpiA operator sites.* + barcode | This paper |
| DTR306 | PAS811 | *Ec spy* + barcode | This paper |
| DTR307 | PAS811 | *Cr TCS7: C. rodentium qseCB  (WP_012907610 and WP_024132939) – P_ygiW_ (to gene locus tag ROD_RS17570)* + barcode | This paper |
