# Supplementary figures and images for: A discovery platform for identification of host-induced bacterial biosensors from diverse sources
Source: Mol Syst Biol. 2025 Jun 9;21(9):1237–62. doi: 10.1038/s44320-025-00123-3 (PMC12405535; doi:10.1038/s44320-025-00123-3)

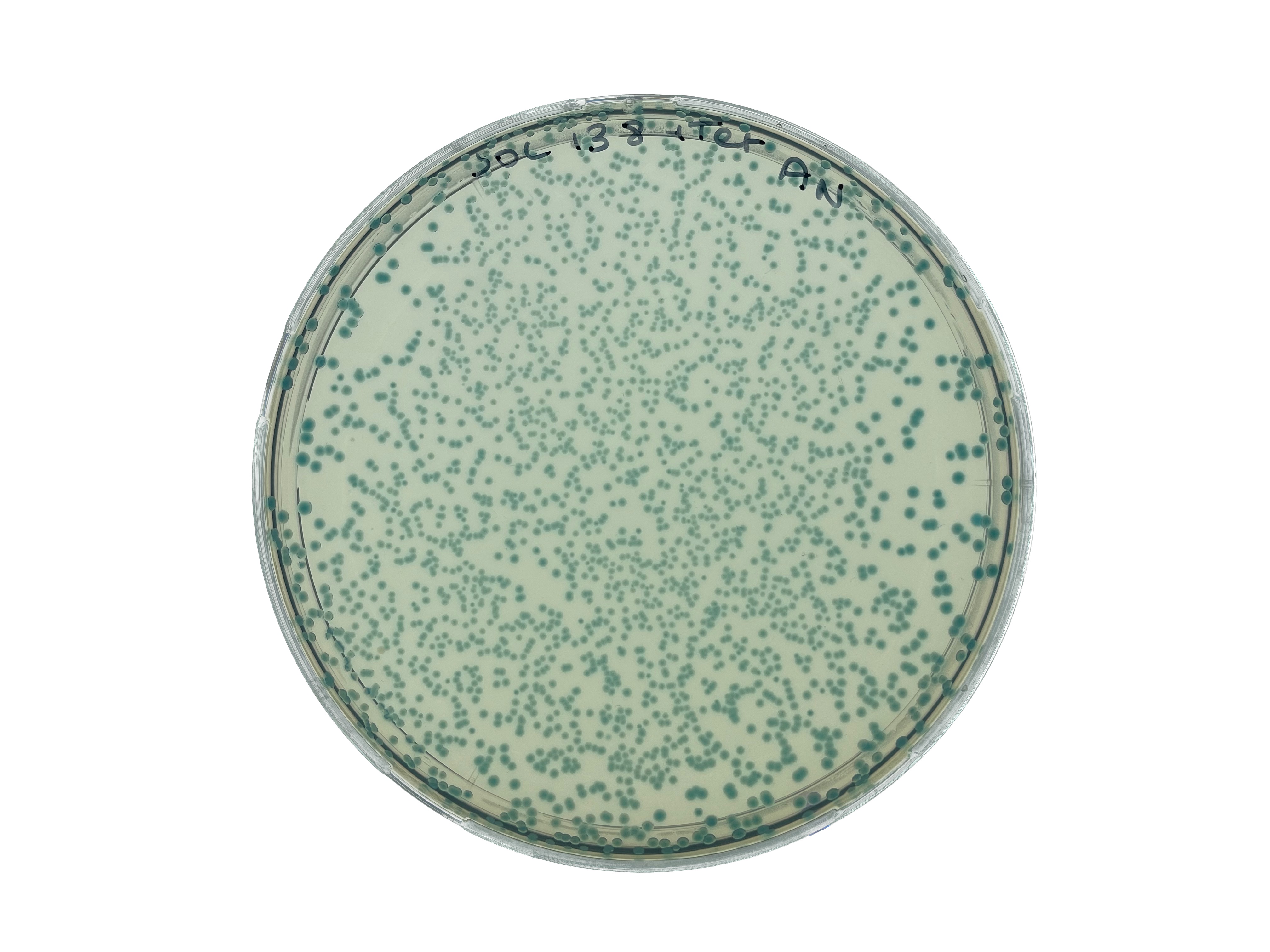

Supplement: Supplementary file 6 — Source data Fig. 2 [file 44320_2025_123_MOESM6_ESM.zip › Figure 2 Source Data/Fig_2D_2.jpeg]

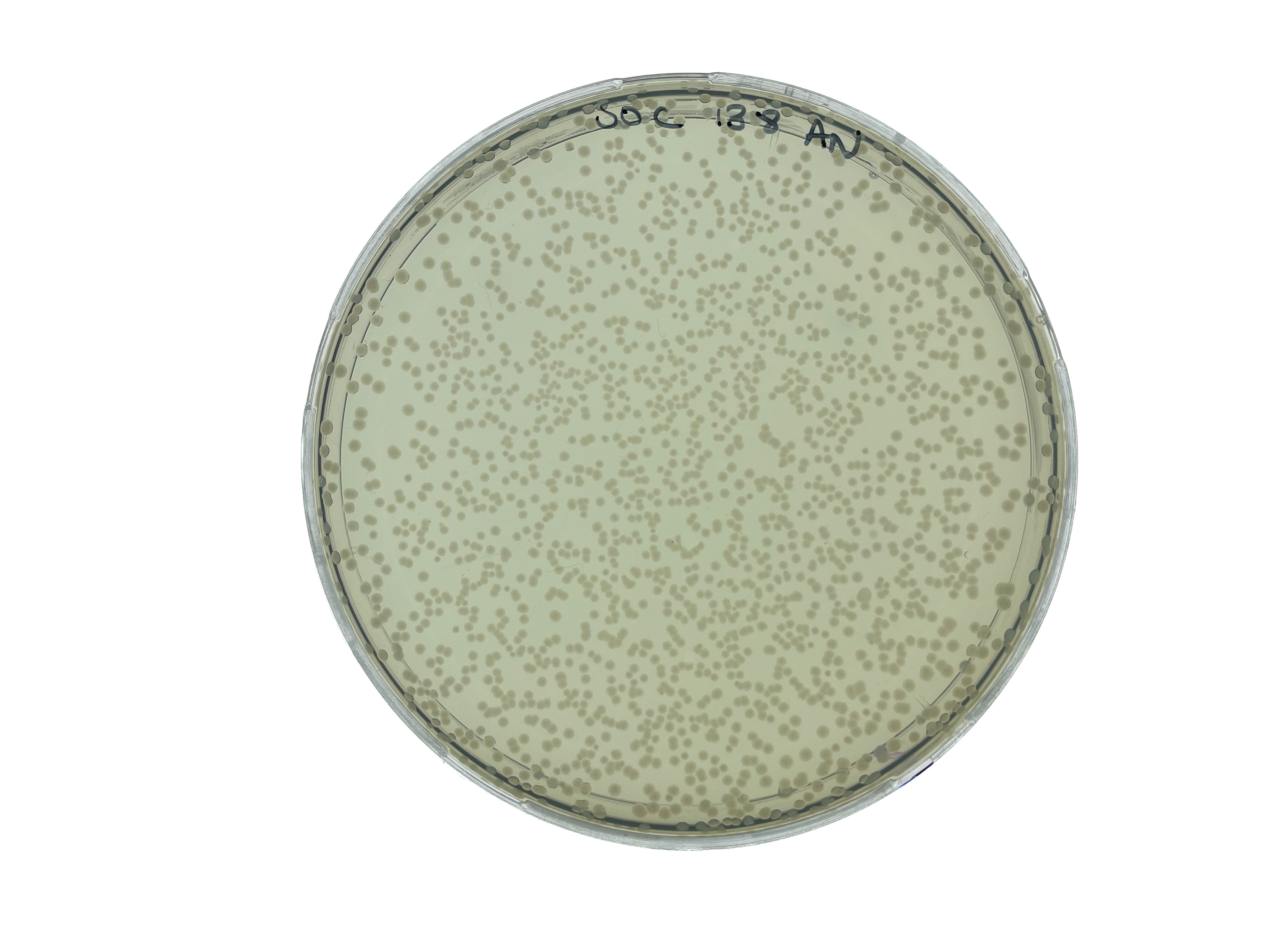

Supplement: Supplementary file 6 — Source data Fig. 2 [file 44320_2025_123_MOESM6_ESM.zip › Figure 2 Source Data/Fig 2D_1.jpeg]
