# Supplementary material for: A discovery platform for identification of host-induced bacterial biosensors from diverse sources
Source: Mol Syst Biol. 2025 Jun 9;21(9):1237–62. doi: 10.1038/s44320-025-00123-3 (PMC12405535; doi:10.1038/s44320-025-00123-3)
Supplement: Supplementary file 10 — Expanded View Figures [file 44320_2025_123_MOESM10_ESM.pdf]

Expanded View Figures

Figure EV1. Library cloning strategy and validation.

(**A**) Libraries were constructed by multiplexed golden gate assembly to create a barcoded plasmid library, which was first transformed into *E. coli* DH5a, then miniprepped and transformed into *E. coli* NGF-1 (PAS811), a murine gut commensal *E. coli* strain carrying the high-throughput memory circuit. Induction of Tn7 genome integration machinery by arabinose and curing of plasmids by multiple growth steps at 42 °C afforded each final pooled library. (**B**) Barcodes were assigned to sensors by a combination of Nanopore long-read sequencing and Illumina short-read sequencing. Lower fidelity long-read data were first aligned to sensor templates, and associated barcode regions were then trimmed and aligned to high confidence barcode sequences that were inferred using Dada2 clustering from Illumina short-read sequences. A barcode was assigned to a sensor if it had both the highest number of trimmed long reads and highest fraction of aligning trimmed long reads normalised by total long reads for the specific sensor. (**C**) Number of assigned barcodes per sensor within libraries 1 and 2. (**D**) Fractions of total reads across all samples of each library's assigned barcodes.

© The Author(s)

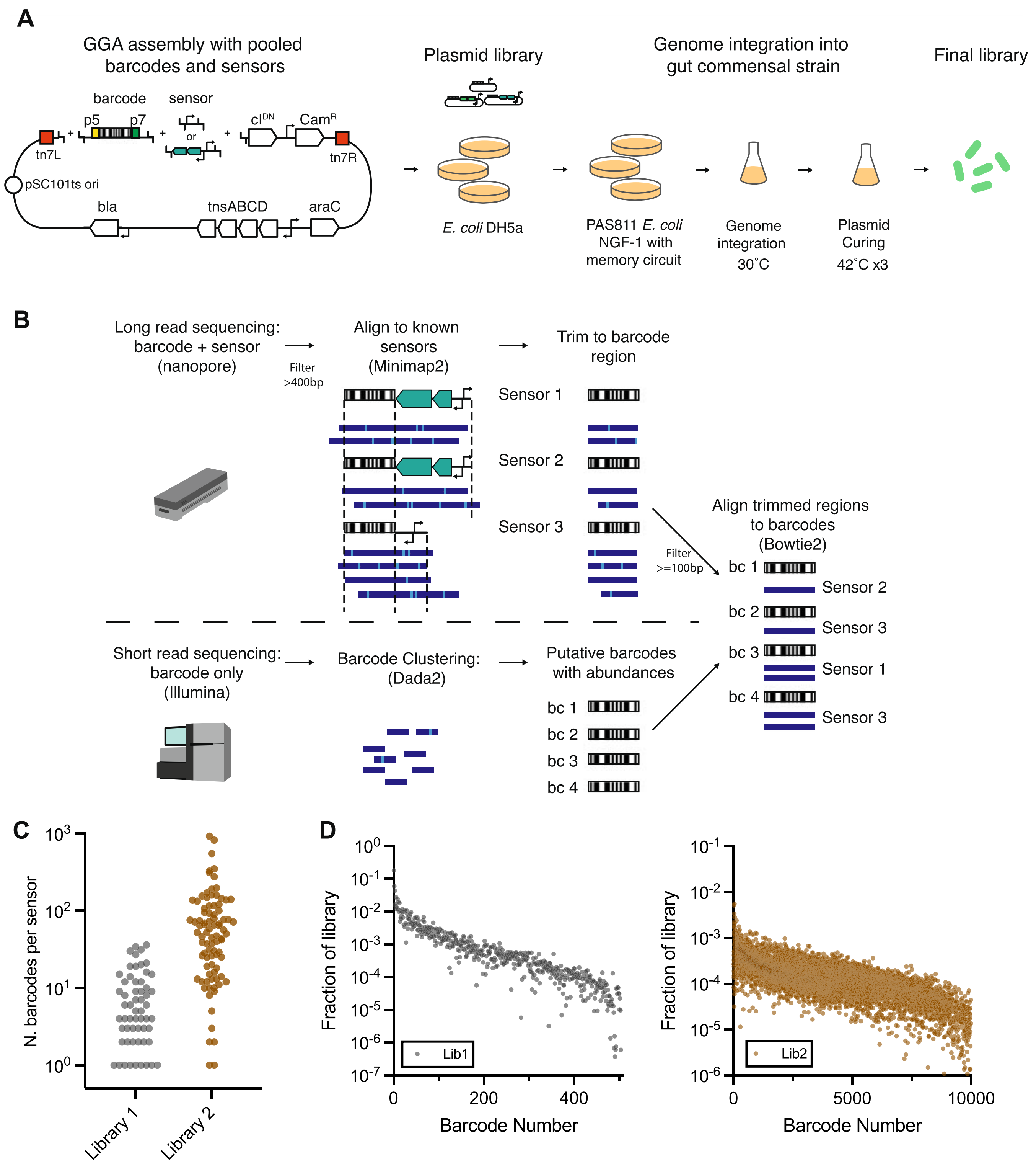
A
GGA assembly with pooled barcodes and sensors
barcode
sensor
p5
p7
or
cI^DN
Cam^R
tn7L
tn7R
pSC101ts ori
bla
tnsABCD
araC
Plasmid library
E. coli DH5a
Genome integration into gut commensal strain
PAS811 E. coli NGF-1 with memory circuit
Genome integration 30˚C
Plasmid Curing 42˚C x3
Final library
B
Long read sequencing: barcode + sensor (nanopore)
Filter >400bp
Align to known sensors (Minimap2)
Trim to barcode region
Sensor 1
Sensor 2
Sensor 3
Align trimmed regions to barcodes (Bowtie2)
Filter >=100bp
bc 1
Sensor 2
bc 2
Sensor 3
bc 3
Sensor 1
bc 4
Sensor 3
Short read sequencing: barcode only (Illumina)
Barcode Clustering: (Dada2)
Putative barcodes with abundances
bc 1
bc 2
bc 3
bc 4
C
N. barcodes per sensor
Library 1
Library 2
D
Fraction of library
Lib1
Barcode Number
Lib2
Barcode Number

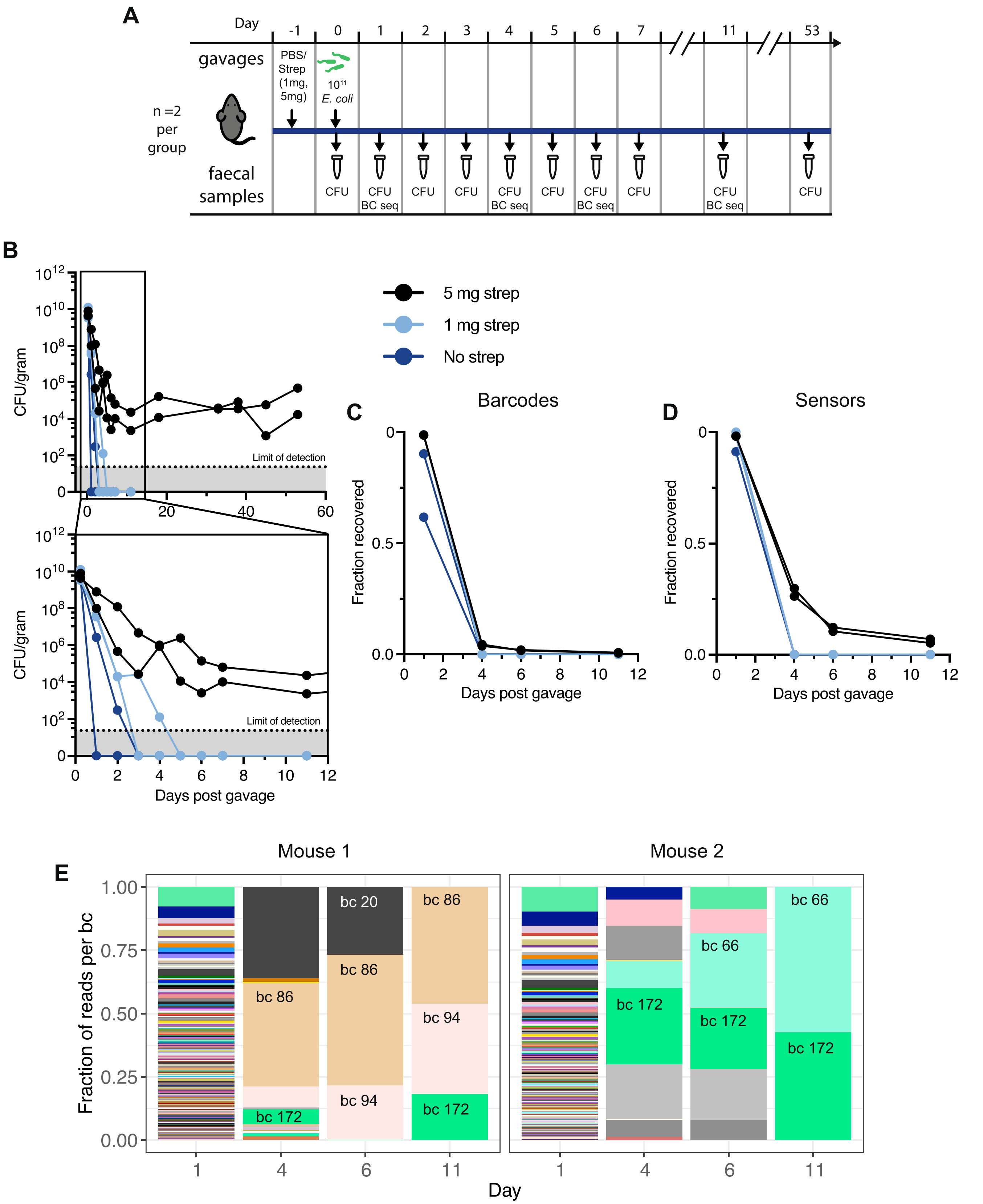
A
Day
-1
0
1
2
3
4
5
6
7
11
53
gavages
PBS/ Strep (1mg, 5mg)
10^{11} E. coli
n =2 per group
faecal samples
CFU
CFU BC seq
B
CFU/gram
Limit of detection
Days post gavage
5 mg strep
1 mg strep
No strep
C
Barcodes
Fraction recovered
Days post gavage
D
Sensors
E
Mouse 1
Mouse 2
Fraction of reads per bc
bc 20
bc 86
bc 94
bc 172
bc 66
Day

Figure EV2. Barcode and strain diversity is maintained over short periods in the murine gut.

(**A**) C57BL/6 mice ($n = 2$ per group) were administered different concentrations of streptomycin (0, 1 or 5 mg) via oral gavage, followed by ~10^{11} Library 1 bacteria the following day. Faecal samples were measured for colonisation by plated CFU counts and for diversity by Illumina barcode sequencing. (**B**) CFU counts of engineered bacteria. (**C**) Fraction of total library barcodes and (**D**) fraction of total sensors recovered across the experiment. (**E**) Relative abundance of each barcode within two mice treated with 5 mg streptomycin. Individual barcodes with high abundances in later timepoints are numbered and highlighted for clarity.

 © The Author(s)

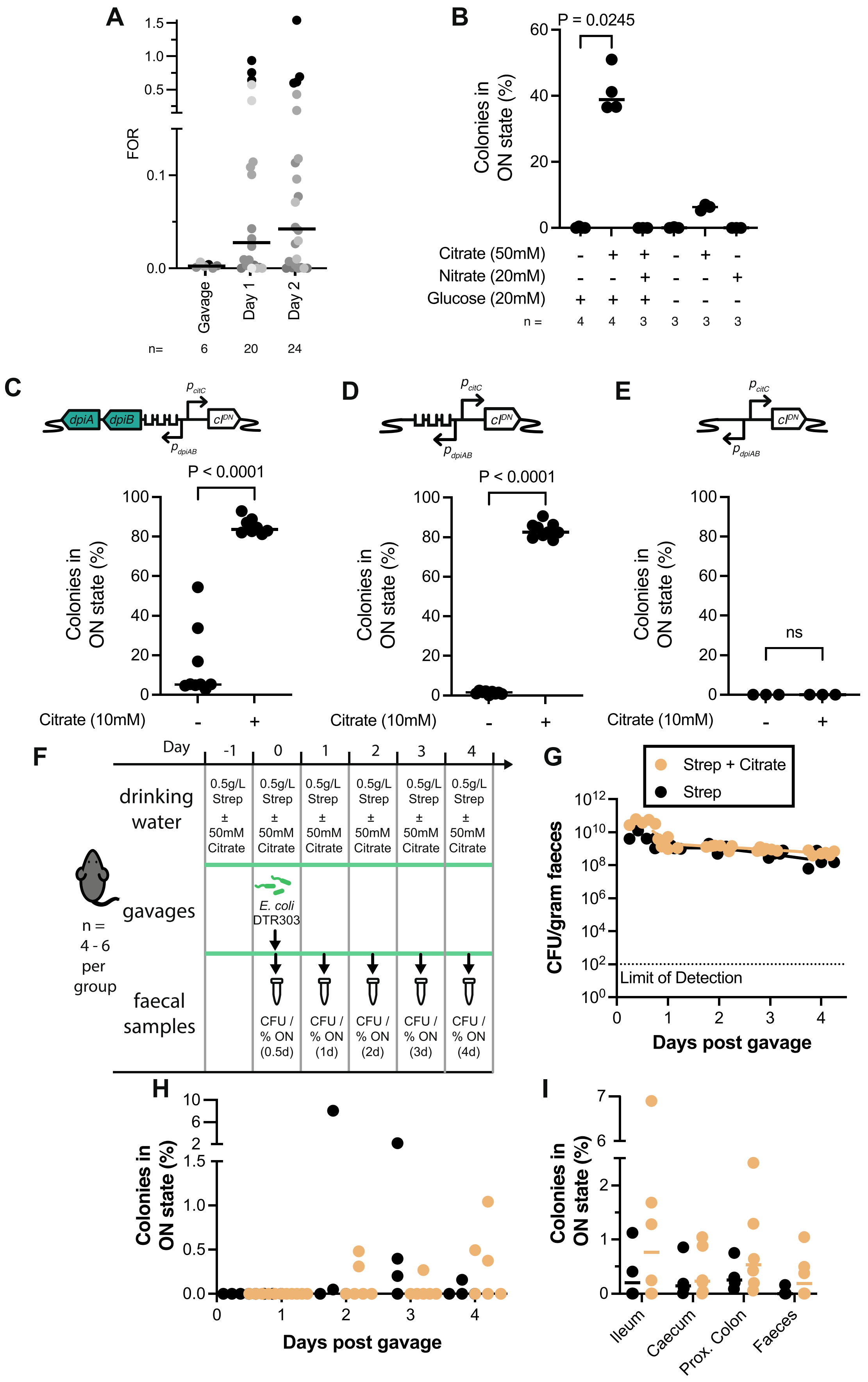
A
FOR
Gavage
Day 1
Day 2
n= 6 20 24
B
P = 0.0245
Colonies in ON state (%)
Citrate (50mM)
Nitrate (20mM)
Glucose (20mM)
n = 4 4 3 3 3 3
C
dpiA
dpiB
P_{citC}
P_{dpiAB}
cI^{DN}
P < 0.0001
Colonies in ON state (%)
Citrate (10mM)
D
P < 0.0001
Citrate (10mM)
E
ns
Citrate (10mM)
F
Day -1 0 1 2 3 4
drinking water
0.5g/L Strep ± 50mM Citrate
gavages
E. coli DTR303
faecal samples
CFU / % ON (0.5d)
CFU / % ON (1d)
CFU / % ON (2d)
CFU / % ON (3d)
CFU / % ON (4d)
n = 4 - 6 per group
G
Strep + Citrate
Strep
CFU/gram faeces
Limit of Detection
Days post gavage
H
Colonies in ON state (%)
Days post gavage
I
Colonies in ON state (%)
Ileum
Caecum
Prox. Colon
Faeces

Figure EV3. Cr TCS2 biosensor response and validation in vitro and in vivo.

(**A**) The response of Cr TCS2 (DpiAB -PcitC) sensors to the gut. Panels show FOR of individual barcodes ($n = 5$–8) in faecal pellets from each mouse ($n = 3$ D1 and $n = 4$ D2) & gavage ($n = 1$) sample passing QC. Shades correspond to unique barcode variants with total datapoints and median response marked. (**B**) Switching of DTR303, an individually cloned Cr TCS2 sensor, grown under in vitro anaerobic conditions in the presence of the regulator levels shown, as measured by indicator plating. Graph shows counts from $n = 3$–4 biological replicates (as labelled) with median marked. Statistics were calculated using a Kruskal-Wallis test with Dunn's multiple comparison correction comparing all groups to the glucose only control. (**C**) Indicator plating of Cr TCS2 sensor variants demonstrated similar response during anaerobic growth with 10 mM citrate between the full-length sensor DTR303 ($n = 9$) and (**D**) a truncation mutant DTR304 lacking the heterologous *C. rodentium* TCS genes dpiA and dpiB ($n = 9$). (**E**) Further truncation of DpiA-binding sites, DTR305, prevented switching ($n = 3$). Graphs in C-E show individual response values with median labelled. Statistics were calculated using a Mann–Whitney test, with significant *P* values shown. (**F**) DTR303 was administered to mice with streptomycin ($n = 4$) and streptomycin + 50 mM citrate ($n = 6$) supplemented drinking water. (**G**) CFU counts of engineered bacteria (**H**) Colony response was measured on selective indicator plates from faecal pellets over 4 days following administration and (**I**) from dissected regions of the gut at endpoint. Graphs show median.

 © The Author(s)

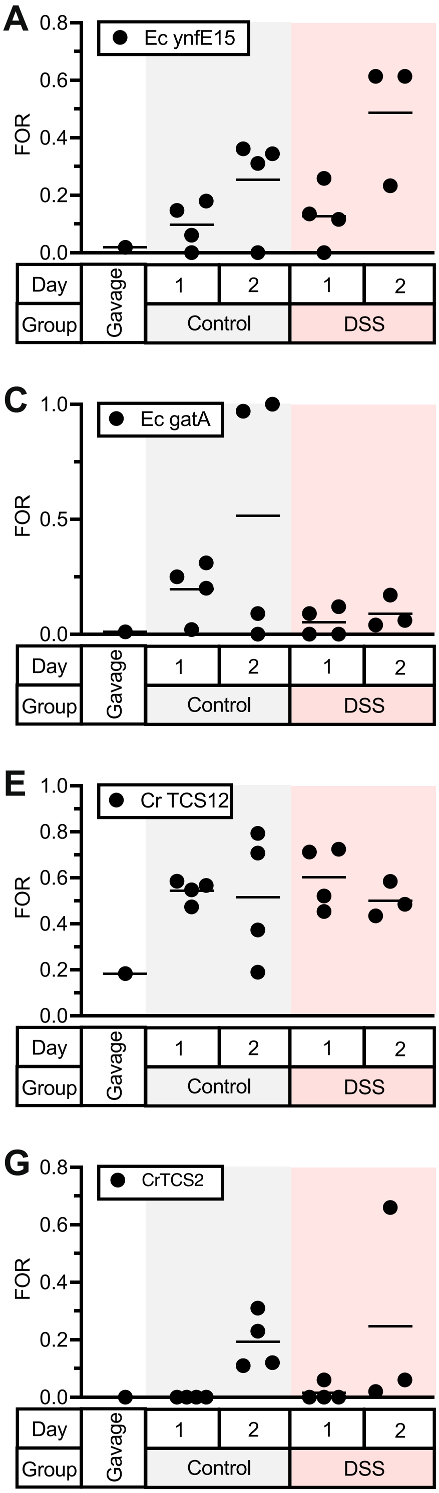

Figure EV4. Screening of Library 1 + 2 identifies in vivo responsive biosensor candidates.

Individual sensor activation on day 1 and 2 post bacterial administration (day 5 and 6 post DSS exposure) for (**A**) Ec ynfE15 control and top in vivo response sensors (**B**) Ec yhcN, (**C**) Ec gatA, (**D**) Cr TCS7, (**E**)) Cr TCS12, (**F**) Ec pspA and (**G**) Cr TCS. Graphs show individual mouse FOR values from datapoints passing sample and sensor QC criteria (for all sensors $n = 4$ for control and DSS day 1, $n = 3$ for DSS day 2 and $n = 1$ for gavage), with mean marked.

© The Author(s)

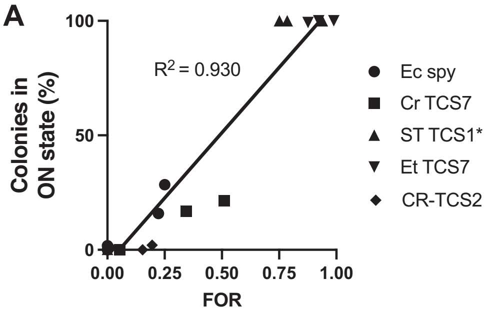

Figure EV5. Library screening results correlate with response of individually tested biosensors.

(**A**) Comparison between FOR from pooled screening and colony counts of individual sensors tested under the same in vivo or in vitro conditions across all validated biosensors in this study. Graph shows mean FOR and mean response for 19 unique strain/condition combinations. A simple linear regression is overlaid.

 © The Author(s)
